# Binge Drinking, Depressive Symptoms, and Sleep Health in Middle-Aged and Older Adults

**DOI:** 10.1093/geroni/igaa057.1373

**Published:** 2020-12-16

**Authors:** Rebecca Lorenz, Samantha Auerbach, Yu-Ping Chang

**Affiliations:** University at Buffalo, Buffalo, New York, United States

## Abstract

Unhealthy alcohol consumption such as binge drinking and depression are common problems among adults. The combined effect of binge drinking and depression might contribute to negative health outcomes, such as accidents, addiction, or sleep problems. Previous evidence has indicated that alcohol consumption differs by age. However, little is known about the association between binge drinking, depression, and sleep health, and how age might play a role in this association. This study aimed to examine the association between binge drinking, depressive symptoms, and sleep health in middle-aged and older adults and characterize any age differences. A total of 5191 middle-aged and older adults from the 2014 Core Survey of the Health and Retirement Survey (HRS) data aged 50 to 80 were included for this study. Binge drinking was defined as the consumption of 5 or more drinks (men) and 4 or more drinks (women) per drinking day. Depressive symptoms were measured using a validated 8-item Center for Epidemiologic Studies Depression Scale. Sleep health was assessed using a composite measure. Age was grouped into middle-aged (50-64.9 years) and older (65-79.9 years) adults. Multiple linear regression analysis was used to examine the associations between variables of interest. Our findings indicated that binge drinking and depressive symptoms negatively influenced sleep health among middle-aged adults, however this relationship was not found in older adults. Clinicians should simultaneously assess problematic alcohol consumption, depressive symptoms, and sleep health. Future research can develop and test age-specific interventions to reduce unhealthy drinking behaviors in middle-aged adults.

